# Chidamide as maintenance after chemotherapy or hematopoietic stem cell transplantation in 27 children with T-cell lymphoblastic leukemia: A real-world prospective study

**DOI:** 10.3389/fmed.2023.1096529

**Published:** 2023-02-02

**Authors:** Xin-Yu Li, Xia-Wei Han, Ke Huang, Ya-Ting Zhang, Hong-Gui Xu, Dun-Hua Zhou, Lu-Hong Xu, Jian-Pei Fang

**Affiliations:** ^1^Department of Pediatrics, Sun Yat-sen Memorial Hospital, Sun Yat-sen University, Guangzhou, China; ^2^Guangdong Provincial Key Laboratory of Malignant Tumor Epigenetics and Gene Regulation, Sun Yat-sen Memorial Hospital, Sun Yat-sen University, Guangzhou, China

**Keywords:** Chidamide, T-cell acute lymphoblastic leukemia, children, chemotherapy, hematopoietic stem cell transplantation

## Abstract

**Background:**

The long-term overall survival of children with T-cell acute lymphoblastic leukemia (T-ALL) is limited to approximately 80–85% because of a high incidence of relapse after achieving remission with intensive chemotherapy and hematopoietic stem cell transplantation (HSCT). Novel treatment strategies inducing long-term remission are needed to improve the outcome. Histone deacetylase inhibitors (HDACis) have been reported to be effective in a series of T-ALL cases. Preclinical studies suggested that T-ALL cells are sensitive to Chidamide, which is a selective HDACi.

**Methods:**

This preliminary clinical study evaluated the efficacy and safety of Chidamide in combination with chemotherapy or post-HSCT for children with T-ALL at a dose of 0.5 mg/kg weight of patient twice per week for at least 6 months.

**Results:**

In total, 27 children with a mean age of 7.88 years were included. The high-risk proportion was 66.7%. After a median follow-up period of 37.8 months (9.5–67.9 months), the overall survival and event-free survival in the patients treated with Chidamide were 94.1 and 95.2%, respectively. All patients except two maintained persistent remission with <0.01% blast cells in minimal residual disease.

**Conclusion:**

The combination therapy with Chidamide in a case series of T-ALL shows the promising clinical efficacy and good safety in children.

**Clinical trial registration:**

https://www.chictr.org.cn/, identifier ChiCTR2000030357.

## Introduction

T-cell acute lymphoblastic leukemia (T-ALL) accounts for approximately 12–15% of all ALL cases in childhood. The long-term overall survival (OS) of children with T-ALL is limited to approximately 80–85% because of a high incidence of relapse after achieving remission with intensive chemotherapy (1–3). Currently, the first-line induction therapy is a combination of intensive chemotherapy of Dexamethasone/prednisone, vincristine, pegaspargase, daunorubicin, 6-mercaptopurine, and cytarabine, while consolidation therapy includes cyclophosphamide, cytarabine, 6-mercaptopurine, pegaspargase, vincristine, nelarabine, and high-dose methotrexate. Progress has been achieved in the nelarabine treatment of T-ALL (4). Lately, venetoclax and bortezomib in combination with chemotherapy has been reported to be effective in relapsed or refractory (R/R) T- ALL (5). However, the complete remission (CR) rate for nelarabine has been only 31–36%. The duration of response was short except in a small proportion of patients who underwent allogeneic hematopoietic stem cell transplantation (HSCT) (6). Consequently, novel treatment strategies for T-ALL that can maintain long-term remission are needed to improve the outcome.

Studies have suggested that high expression of Histone deacetylase (HDAC) in childhood T-ALL is associated with poor prognosis, which indicates that HDAC inhibitors (HDACis) may be used to treat T-ALL (7). HDACis have been recommended for relapsed or refractory T-cell lymphoma and have been reported to be effective in cases (8). HDACi reverses the inhibition of the transcription of tumor-suppressor and related genes, which is caused by HDAC acetylating the Lysine residues on histone proteins (9, 10). It has been approved by the National Medical Products Administration for the treatment of relapsed and refractory peripheral T-cell lymphoma, and it has been approved by the FDA for clinical study (8, 11). Chidamide in combination with chemotherapy has been proved to be effective in R/R T lymphoblastic lymphoma/leukemia in adults and young adolescents (8). Furthermore, Chidamide does not significantly increase the adverse effects of chemotherapy (12).

Based on these rationales, for the treatment of children with T-ALL at our institution, Chidamide has been added to the chemotherapy and for post-HSCT relapse prophylaxis. Here, we report the clinical efficacy and safety of the Chidamide in a case series of 27 patients with first-time diagnosed T-ALL.

## Materials and methods

### Patient population

From July 1, 2016, to May 31, 2021, 235 children were diagnosed with T-ALL in the SCCLG-2016 cooperation group. The SCCLG-2016 ALL protocol (Supplementary Information 1) was adopted (ChiCTR2000030357). 27 patients, who treated with Chidamide (Epidaza^®^), were included in the present study. Patients provided informed consent to receive Chidamide. We excluded patients who received Chidamide for <24 weeks and who abandoned treatment at any point of therapy without achieving any endpoint. The research protocol was approved by the ethics committee of Sun Yat-sen Memorial Hospital of Sun Yat-sen University. All data were prospectively collected and analyzed.

### Diagnosis, response assessment and risk stratification

The diagnosis of T-ALL was based on morphological French-American-British criteria: negative staining for myeloperoxidase or Sudan black and immunophenotypical expression of T- cell surface antigens CD7 or CD2, often with CD5, CD3, CD4, or CD8 in more than 20% of blast cells and nuclear deoxynucleotide transferase or intracytoplasmic CD3 in more than 10% of blast cells. Unequivocal cases were reviewed centrally. Immunophenotyping was performed according to the European Group for the Immunological Characterization of Leukemias or World Health Organization 2008 criteria.

CR was defined as bone marrow blast cells ≤5%, absence of extramedullary disease and recovery of peripheral blood counts (absolute neutrophil count ≥ 1.0 × 10^9^/L and platelets ≥ 100 × 10^9^/L). Minimal residual disease (MRD) in bone marrow aspiration specimens was assessed by multiparameter flow cytometry with a sensitivity of 0.01%. flow cytometry MRD was analyzed according to a previous French multicenter study for pediatric and adult ALL. MRD was analyzed by Kaluza software (Beckman Coulter, United States) or CellQuest software (BD Biosciences, Franklin Lakes, NJ, USA). Reagents were provided by BD Biosciences (Becton, Dickinson, China) and Beckman Coulter Commercial Enterprise (China) Co., Ltd.

After the seven-day prednisone test and dexamethasone-based four-drug induction (Supplementary Information 1), patients with T-ALL were classified into the intermediate-risk (IR) and high-risk (HR) groups based on the response to early treatment. Central nervous system leukemia (CNSL) was diagnosed when white blood cell count (WBC) was higher than 5/mL with blasts were found in cerebrospinal fluid or when clinical symptoms of cranial nerve palsy, brain or eye involvement, or hypothalamic syndrome were found. HR criteria were poor prednisone response with the presence of ≥1 × 10^9^ blasts/L in PB on Day 8 after seven-day prednisone monotherapy and one dose of triple intrathecal therapy (TIT), no partial bone marrow remission (M3 [≥25% blasts] or MRD ≥ 10%) on Day 15, no bone marrow remission (M2 [5–25% blasts]/M3, MRD ≥ 1%) after induction phase 1 (Day 33), and/or positive *MLL* gene rearrangement, low diploidy, iAMP21 or *IKZF1* deletion, *MEF2D* rearrangement and *TCF3-HLF* gene fusion, and/or mediastinal tumor lesion evaluated on Day 33 without reduction to 1/3 of the original tumor size or tumor still exists before consolidation therapy. The remaining patients were considered IR.

### Treatment

The treatment protocols for T-cell ALL were based on the ALL-IC-BFM 2002 (13), and one dose of 1000 mg/m^2^ of Cyclophosphamide (CTX) was added to the traditional BFM induction therapy (Supplementary Information 1). Some modifications in systemic therapy and, mostly, in the CNSL prophylactic therapy were made in both the IR group and the HR group. Prophylactic cranial radiotherapy was not recommended. Central nervous system (CNS) involvement was treated with additional TIT. Cranial radiotherapy of 18 Gy was indicated in TIT refractory CNSL only.

Transplantation conditioning regimens for T-ALL were myeloablative, which included: Busulfan 12.8 mg/kg, Fludarabine 120–150 mg/m^2^, Cyclophosphamide 120 mg/kg, and Semustine 250 mg/m^2^ (or cranial radiotherapy of 9 Gy). Total-body irradiation (TBI) was recommended in peripheral blood stem cell (PBSC) match sibling donor transplantation.

Chidamide was used at a dose of 0.5 mg/kg patient weight every 4 days in combination with chemotherapy for patients with T-ALL undergoing maintenance therapy or started at 4–8 weeks post-HSCT for 2 years.

### Statistical analyses

Overall survival was measured as the time from the diagnosis of T-ALL to death or last follow-up. OS was calculated from diagnosis to death (from any cause). Event-free survival (EFS) was measured as the time from achieving CR to relapse, treatment related death or last follow-up. The “event-free” duration was defined as the time from diagnosis to the date of treatment-related death or relapse or until the date of the last contact in CR. For all analyses, patients lost to follow-up were censored at the time of their withdrawal. The survival curves were analyzed using the Kaplan–Meier method.

Follow-up data were updated as of March 2022. Toxicity was graded based on the modified Common Terminology Criteria for Adverse Events (CTCAE) v5.0. Statistical analysis was performed by using SPSS 23.0 (SPSS Institute, Cary, NC, USA). *P* < 0.05 was considered to indicate statistical significance.

## Results

### Patient characteristics

In total, 27 patients were treated with Chidamide, of whom 12 started post-HSCT and 15 received Chidamide during post-remission maintenance therapy. The median follow-up period was 37.8 months (9.5–67.9 months). 18 patients were not older than 10 years old. Male to Female ratio was 8: 1. Three of them were diagnosed with CNS involvement and stratified as HR at the first diagnosis. According to the risk stratification criteria, 18 patients were treated according to the protocol of HR T-ALL, among which 12 patients received HSCT. The characteristics of individual patients treated with Chidamide are shown in Table 1.

**TABLE 1:** Patient demographic and clinical characteristics.

Case number	Sex	Age	ETP-ALL or not	Risk stratification	Prednisone test	CNS involvement	Karyotype	Gene variants	HSCT	Relapsed
1	1	13.4	No	HR	1	No	46, XY, inv(9)(p12q13)c[7]	(–)	No	No
2	1	6.4	Yes	HR	2	No	46, XY[5]	(–)	Yes	No
3	1	1.8	No	HR	2	No	46, XY[11]	(–)	No	No
4	1	10	No	HR	1	No	46, XY[4]	(–)	Yes	No
5	1	5.1	No	IR	1	No	46, XY[3]	SIL/TAL1(+)	No	No
6	1	6.1	No	HR	2	No	46, XY[20]	(–)	No	No
7	2	4.2	No	HR	2	No	46, XX[17]	(–)	No	No
8	1	11.3	No	HR	2	No	46, XY[5]	MLL/AF6(+)	Yes	No
9	1	3.8	No	HR	2	No	46, XY[6]	SIL/TAL1(+), P16(+)	Yes	No
10	1	7.7	No	HR	2	No	46, XY, add(2)(q33)[5]/46, XY[10]	(–)	No	No
11	1	12.2	No	IR	1	No	46, XY[1]	P16(+)	No	No
12	1	12.1	Yes	HR	1	Yes	46, XY[20]	(–)	Yes	Yes
13	1	9.4	No	IR	1	No	46, XY, der(9)(p21;p23)[5]/46, XY[5]	(–)	No	No
14	1	8.9	No	HR	1	No	46, XY, der(9)(q12;q31),del(13) (q13;q31), ?−8,?+12[cp9]/46, XY[11]	NOTCH1	No	No
15	1	12	No	HR	1	No	46, XY, t(11;14)(p15;q11)[5]/46, XY, [5]	NOTCH1, FBXW7	No	No
16	1	7.1	No	HR	1	No	46, XY[20]	(–)	Yes	No
17	1	5.3	No	IR	1	No	46, XY[20]	(–)	No	No
18	1	9.2	No	HR	2	No	46, XY[8]	BRAF, NOTCH1	Yes	No
19	1	13.7	No	HR	1	Yes	46, XY[15]	P16(+)	Yes	No
20	1	2.4	No	IR	1	No	NA	NOTCH1	No	Yes
21	1	7	No	HR	2	No	46, xy[16]	HOX11	Yes	No
22	2	7.3	No	IR	1	No	46, xx, 6p-[6]/46, xx[5]	(–)	No	No
23	1	11.4	No	IR	1	No	46, XY, del(9)(p10)[10]	(–)	No	No
24	2	8.9	No	HR	2	No	46, XX	SIL-TAL1, FBXW7, NOTCH1	Yes	No
25	1	3.2	No	IR	1	No	46, XY	(–)	No	No
26	1	13.5	No	IR	1	Yes	46, XY, t(1:16)(p31:q22), del(9)(p21), del(9)(p21)[4]/46, XY[6]	FBXW7 NOTCH	Yes	No
27	1	13.3	No	HR	3	No	46, XY, del(9)(p21.3)	NOTCH1, FBXW7, PTEN, P16(+)	Yes	No

NA, not available; Sex (1 = male, 2 = female); Prednisone test (1 = GPR, 2 = PPR, and 3 = not evaluated); GPR, good prednisone response; PPR, poor prednisone response; CNS, central nervous system; HR, high risk group; IR, intermediate risk group; HSCT, allogeneic hematopoietic stem cell transplantation; P16(+), P16 deletion.

### Risk-stratified outcomes

**FIGURE 1:**
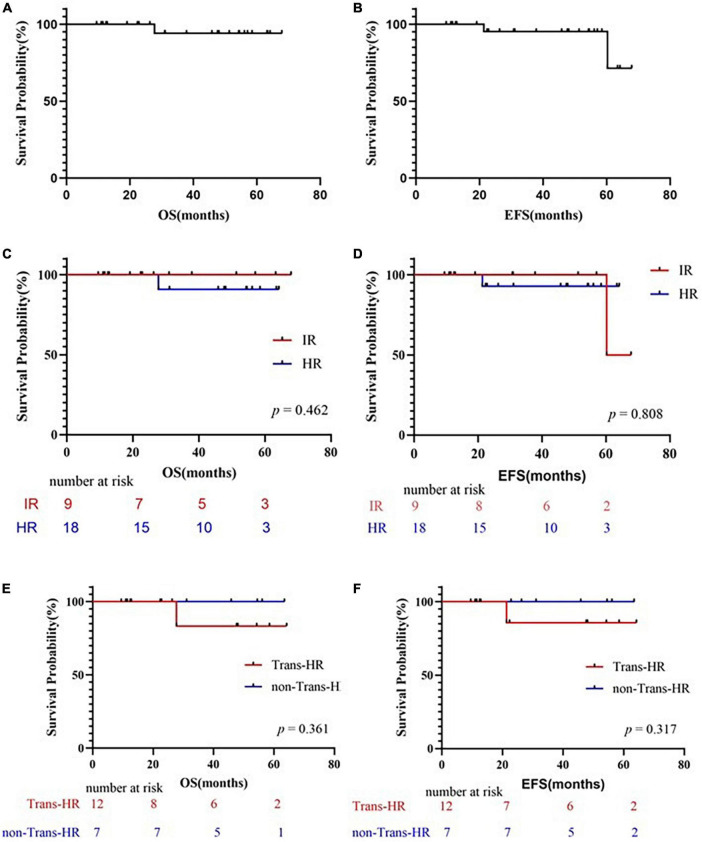
Survival curves. **(A)** Probability of overall survival (OS) for patients treated with Chidamide. **(B)** Probability of EFS for patients treated with Chidamide. **(C)** Comparing the probability of OS for patients treated with Chidamide in IR group and HR group. **(D)** Comparing the probability of EFS for patients treated with Chidamide in IR group and HR group. **(E)** Comparing the probability of OS for patients treated with or without HSCT. **(F)** Comparing the probability of EFS for patients treated with or without HSCT. OS, overall survival; EFS, event-free survival; IR, intermediate-risk; HR, high-risk; HSCT, hematopoietic stem cell transplantation; Trans, treated with HSCT; non-Trans-HR, HR but not treated with HSCT.

Up to the observation time points, 25 of the children receiving Chidamide were in continuous remission. The survival curves are shown in Figure 1. At the end of the 36-month follow-up period, the OS rate was 94.1%, while the EFS rate was 95.2%. In the IR group, both the OS and EFS rates of patients with Chidamide were 100%. One IR patient relapsed in central nervous system and bone marrow subsequently in 30 months after the end of chemotherapy. In the HR group, the OS rates of patients with Chidamide was 90.9%, while the EFS rate was 92.9%. One ETP-ALL patients got relapse in 14 months post-HSCT and deceased in 4 months later.

### HSCT management and outcomes

Most of the HSC donors were unrelated donors (UDs; 9/12; 75%). One was HLA-matched unrelated donors (MUDs), and 8 were mismatched unrelated donors. Two donors were matched sibling donors (MSDs). One donor was haploidentical related donors. The sources of HSCs used were cord blood (*n* = 7), peripheral blood stem cell (*n* = 4), and mixed graft (*n* = 1, P27). The conditioning regimens (Table 2) were classified as myeloablative regimens. TBI was used in three children, which accepted MSD transplantation (*n* = 2) or MUD transplantation (*n* = 1) (Table 2). Five cases of severe conditioning toxicity, not related to Chidamide, were reported: veno-occlusive disease (P24), profuse diarrhea (P19), gastrointestinal bleeding (P24), septicemia and subsequent acute cardiac insufficiency (P24), hepatic toxicity (P24), and grade III to IV hemorrhagic cystitis (P8, 24, 27), severe pneumonia (P12), peripheral neuritis after cyclophosphamide (P19, 27). Severe conditioning regimen adverse effects were well controlled and non-fatal. Graft failure was not reported. Donor lymphocyte infusion was not used among the included patients. TIT was not given post-HSCT

**TABLE 2:** Hematopoietic stem cell transplantation (HSCT) management and outcomes of the 12 high-risk patients.

Patients	HLA match	Source	MNC (×10^7^ cells/kg for CB; 10^8^ cells/kg for PBSC)	Conditioning regimen	Day ANC > 500	GVHD prophylaxis	Donor chimerism (%)	GVHD (acute/chronic)	Status
2	10-Oct	PBSC, sibling	10.5	TBI 9Gy – Cy-VP-16- semustine	11	CsA + MMF + MTX	100	No/No	Disease free and alive
4	10-Oct	PBSC, sibling	10.3	TBI 9Gy – Cy-VP-16- semustine	12	CsA + MMF + MTX	100	No/Yes	Disease free and alive
8	10-Oct	CB	3.7	Bu-Cy-Flu-TBI 14.4Gy	17	CsA + MMF	100	Yes/Yes	alive
9	09-Oct	CB	9.1	Bu-Cy-Flu- semustine	17	CsA + MMF	100	Yes/No	Disease free and alive
12	07-Oct	CB	5	Bu-Cy-Flu-Dec- semustine	19	CsA + MMF	100	Yes/Yes	Relapsed and dead
16	08-Oct	CB	3.3	Bu-Cy-Cla-Ara-C- semustine	22	CsA + MMF	100	Yes/Yes	Disease free and alive
18	09-Oct	CB	4	Bu-Cy-Cla-Ara-C- semustine	14	CsA + MMF	100	Yes/No	Disease free and alive
19	08-Oct	CB	4.7	Bu-Cy-Cla-Ara-C- semustine	17	CsA + MMF	100	Yes/Yes	Disease free and alive
21	09-Oct	PBSC, unrelated	11.3	Cy-ATG-VP-16- semustine	14	CsA + MMF + MTX	100	Yes/No	Disease free and alive
24	08-Oct	CB	3.8	Bu-Cy-Cla-Ara-C- semustine	18	CsA + MMF	100	Yes/No	Disease free and alive
26	09-Oct	PBSC MMUD	12.67	Cy-ATG-VP-16- semustine	11	CsA + MMF + MTX	100	Yes/Yes	Disease free and alive
27	07-Oct	BM + PBSC Haploid father	11.6	Bu-Cy-ATG-Cla-Ara-C- semustine	13	CsA + MMF + MTX	100	Yes/No	Disease free and alive

ANC.500, absolute neutrophil count.500/mm^3^; Ara-C, cytarabine; ATG, antithymocyte globulin; BM, bone marrow stem cell graft; Bu, busulfan; CB, cord blood stem cell graft; Cla, cladribine; CsA, cyclosporin A; Cy, cyclophosphamide; Flu, fludarabine; GVHD, graft versus host disease; haplo-id, haplo-identical donor; MMF, mycophenolate mofetil; MNC, mononuclear cell count; MTX, methotrexate; PBSC, peripheral blood stem cells; TBI, total body irradiation; VP-16, etoposide.

In total, 12 patients accepted HSCT at their first complete remission, and accepted Chidamide post-HSCT regularly. The overall survival and event free survival rate were 83.33 and 85.7% at 64.2 months after transplantation (Figures 1E, F), respectively, with a median follow-up of 27.8 months in surviving children (range, 9.5–64.2 months). One deceased patient (P12) suffered from CNSL relapse at the sixth month post-HSCT, which proceeded to bone marrow relapse at the end. This relapsed patient died before second remission and did not receive second HSCT. Infection was a major complication in the population accepted HSCT. Bacterial, fungal, or virus infections occurred after HSCT in 42% (5/12) of the patients with successful engraftment, but only 2 of these patients had infections during Chidamide therapy after HSCT (P8: lung invasive fungal disease; P12: sinusitis and non-tuberculosis mycobacteria pneumonia). 10 out of the 12 transplanted patients suffered from acute graft versus host disease (GVHD) (83.3%), but only 2 of them had severe GVHD. The incidence of chronic GVHD was 50% (6/12).

### Treatment-related adverse events

No death was reported over the first year of Chidamide combination therapy and subsequent deaths were all related to disease progression. The adverse events observed during treatment were nausea or vomiting (*n* = 6), neutropenia (*n* = 9), thrombocytopenia (*n* = 9), renal impairment (*n* = 1) and infection (Herpes Zoster, *n* = 1). The adverse events were all reversible when administration of Chidamide was stopped, and Chidamide therapy was continued with previous doses until relapse, or the end of maintenance therapy, or 2 years post-HSCT.

## Discussion

In this study, we prospectively investigated the safety and efficacy of the combination of Chidamide with chemotherapy post-remission or post-HSCT in pediatric T-ALL patients. We show that the addition of Chidamide to the traditional regimen has promising effects in relapse prophylaxis. The most important findings in this study are (i) that the relapse rate in the high-risk group with or without HSCT was reduced and (ii) that a few severe adverse effects were found in the Chidamide group for post-HSCT and post-remission maintenance in pediatric T-ALL.

The long-term survival of patients with T-ALL relapse has been lower than 40%, even with intensive chemotherapy or post-HSCT (14). The significance of relapse prophylaxis has been recognized. However, the effectivity of relapse prophylaxis is limited, and the optimal strategy is controversial. The results in this study indicate the efficacy of Chidamide in relapse prophylaxis.

Preclinical study results indicated the promising efficacy of Chidamide. Chidamide may regulate immune function (15) and increase the sensitivity of T-ALL cells to chemotherapy drugs independent of *NOTCH* mutation (16). The mechanistic investigations indicated the higher efficacy of HDACi in HR and R/R T-ALL. In clinical studies, Chidamide in combination with chemotherapy has been proved effective in R/R T-cell lymphoblastic lymphoma/leukemia in adults and young adolescents (8, 11, 12). The efficacy of Chidamide in peripheral T-cell lymphoma suggests that Chidamide may play a role in more immature T-LBL/ALL (17, 18). In our study, all T-ALL patients except one showed persistent remission after maintenance chemotherapy resulting in a reduced relapse rate, similar to its effects in AML or R/R T-cell lymphoblastic lymphoma/leukemia studies (19), indicating a promising relapse prophylaxis option for T-ALL (20). The relapse rates in the IR and HR groups were similar. The results support that Chidamide can be included in remission prevention regimens and reduce the intensity of chemotherapy for T-ALL in the future (8, 21, 22). High-risk and refractory T-ALL are more likely to benefit from Chidamide combination chemotherapy.

Our results showed a lower incidence of relapse than previous studies in both adults and children. In the last decade, EFS and OS rates for T-ALL children in several large cohorts have been about 73–80% (23–25). For those who survived the early intensive chemotherapy, relapse rate and HSCT related mortality were still high (23–25). In this study, the EFS rate for T-ALL patients was up to 95.2 ± 4.6%, which was much higher than in previous reports. The exclusion of early fatal cases irrelevant to Chidamide was one important factor for this result. The most important risk factor for early mortality in our center was fatal complications during intensive chemotherapy in the first 6 months, especially in the HR group. The Chidamide group had a higher proportion of HR and HSCT acceptance than general T-ALL population, but Chidamide increased neither relapse related mortality nor HSCT related mortality. The good OS and EFS results in this study indicate the promising efficacy and safety of Chidamide.

There is concern that Chidamide cannot penetrate the brain blood barrier to the cerebrospinal fluid, which makes it difficult to prevent CNSL relapse through Chidamide. According to previous studies, multiple triple drugs intrathecal injections during intensive chemotherapy and maintenance chemotherapy, as adopted in our protocol, are the primary and reliable effective CNSL prophylaxis.

In previous study, for T-ALL patients who underwent alternative donor HSCT using a mismatched, umbilical cord blood, or haploidentical donor, OS rate at 5 years was only 34%, while non-relapse mortality and relapse was 26 and 41%, respectively (26), even the participation of TBI in conditioning regimens. Lately, it was reported that the 5-year leukemia free survival of T-ALL patients receiving HSCT at first complete remission was as high as 73.5%. However, once relapse occurred, the 5-year leukemia-free survival (LFS) was only 44.4% even if second remission was attained (27). If they accepted umbilical cord blood transplantation (UCBT) at first complete remission, TBI-based myeloablative conditioning presented LFS rate of 85.4%, as it reduced the risk of relapse, while other conditioning regimens prognosed worse (62.2%) (27). So, the LFS after UCBT for T-ALL were 65.8–85.4% (27, 28). The key points of long-term survival would be proper conditioning regimens, transplantation at the first complete remission and relapse prophylaxis. Donor cell transfusion would be an effective option, except for those with GVHD or those accepted UCBT, just like patients in this study. There is a new trend that post-HSCT antitumor medication participates in the relapse prophylaxis of leukemia therapy. Molecular targeted drugs, like tyrosine kinase inhibitors, demethylation therapy, Bcl-2 inhibitors, have been recommended (29–31). However, for most of T-ALL, there is no recommended medicine for post-HSCT relapse prophylaxis. Chidamide has been a new attempt, which has showed great potential according to the low post-HSCT relapse rate and the low HSCT related complications incidence.

With respects to observation end-points, since the follow-up period is now only 3 to 4 years, there is a possibility that some patients have not achieved the end-point of relapse. However, according to the Berlin–Frankfurt–Munster (BFM) protocol-based CCLG-ALL2008 protocol in China, relapse cases were reported mostly ultra-early (65%, in the first 18 months after first diagnosis), and early (30%, in the second 18 months after first diagnosis). Only less than 5% of relapse cases occurred after more than 36 months (32). These previous data indicated that the follow-up period of the present study is effective for observation for relapse rates. Further studies should include prolonged observation periods.

The dose of Chidamide was 0.5 mg/kg in this study. We adopted this dosage from previous studies, which included children younger than 18 years old (8, 12). The plasma concentration of Chidamide was not monitored. The adverse events observed during treatment were nausea or vomiting, neutropenia, thrombocytopenia, renal impairment and infections, which were similar to previous reports. It did not significantly increase the adverse effects of maintenance chemotherapy. Since the patients’ compliance was good, by monitoring WBC, hemoglobin levels, and platelet count, we adjusted the dosages of Chidamide. When grade 3 or worse bone marrow suppression occurred, we reduced the dosage or discontinued treatment. WBC between 2 × 10^9^/L and 4 × 10^9^/L is safe and efficient for maintenance therapy.

There are some limitations in this study. Considering the limited number of cases, a larger number of patients should be enrolled to further analyze the efficacy of Chidamide in T- ALL relapse prophylaxis. Furthermore, it was difficult to analyze the efficacy differences between subgroups based on, for example, gene mutation, risk stratification, and early T-cell precursor ALL (33). To further analyze the efficacy of Chidamide in different T-ALL subtypes, we need more large-scale prospective data.

In conclusion, we reported a series of cases of firstly diagnosed T-ALL who received Chidamide during maintenance regimens or post-HSCT with a remission rate of 95% in more than 3 years, showing promising efficacy in relapse prophylaxis. Maintenance regimens containing Chidamide are well tolerated in T-ALL children. The benefit of Chidamide in prolonged EFS deserves further study and confirmation by large-scale prospective clinical trials.

## Data availability statement

The raw data supporting the conclusions of this article will be made available by the authors, without undue reservation.

## Ethics statement

The studies involving human participants were reviewed and approved by the Ethics Committee of Sun Yat-sen Memorial Hospital of Sun Yat-sen University. Written informed consent to participate in this study was provided by the participants’ legal guardian/next of kin.

## Author contributions

J-PF designed the study. X-YL and X-WH analyzed the data and wrote the manuscript. X-YL, X-WH, and KH contributed to the retrieval and analysis of essential data. All authors contributed to the charts, critical revision, and final approval of the manuscript.
