# Supplementary material for: Chidamide as maintenance after chemotherapy or hematopoietic stem cell transplantation in 27 children with T-cell lymphoblastic leukemia: A real-world prospective study
Source: Front Med (Lausanne). 2023 Feb 2;10:1096529. doi: 10.3389/fmed.2023.1096529 (PMC9932021; doi:10.3389/fmed.2023.1096529)
Supplement: Supplementary file 1 [file Table_1.DOCX]

Supplementary Information I. The protocol applied for T-ALL induction and consolidation

| Risk Group | Phase of therapy | Drug/Dose | Day |
| --- | --- | --- | --- |
| IR/HR | Induction Phase 1 | Prednisone 60 mg/m^2^ PO | D1-7 |
|  |  | Dexamethasone 6 mg/m^2^ PO | D8-37 |
|  |  | Vincristine 1.5 mg/m^2^ (max.2 mg) IV | D8,15,22,29 |
|  |  | Daunorubicin 30 mg/m^2^ PI over 1 h | D8,15,22,29 |
|  |  | PEG-asparaginease 2500 mg/m^2^ (max. 3750 IU) IM | D9,23 |
|  |  | Cyclophosphamide 1000 mg/m^2^ PI over 1 h | D10 |
|  |  | IT | D1,8,15,22,33 |
|  | Induction Phase 2 | Cyclophosphamide 1000 mg/m^2^ PI over 1 h | D1,29 |
|  |  | Cytarabine 75 mg/m^2^ IV | D3-6,10-13,31-34,38-41 |
|  |  | 6-mercaptopurine 60 mg/m^2^ PO | D1-14, 29-42 |
|  |  | PEG-asparaginease 2500 mg/m^2^ (max. 3750 IU) IM | (IR only) D15,43 |
|  |  | IT | D3,31 |
|  | Consolidation |  |  |
| IR |  | 6-mercaptopurine 25 mg/m^2^ PO | D1-56 |
|  |  | Methotrexate 5000 mg/m^2^ PI over 24 h | D8,22,36,50 |
|  |  | IT | D8,22,36,50 |
| HR | Block1 | Dexamethasone 20 mg/m^2^ PO/IV | D1-5 |
|  |  | Vincristine 1.5 mg/m^2^ (max. 2mg) IV | D1,6 |
|  |  | Methotrexate 5000 mg/m^2^ PI over 24 h | D1 |
|  |  | Cyclophosphamide 200 mg/m^2^ PI over 1 h | D2-4 (five doses, 12 h interval) |
|  |  | Cytarabine 2000 mg/m^2^ IV PI over 3 h | D5 (two doses, 12 h interval) |
|  |  | PEG-asparaginease 2500 mg/m^2^ (max. 3750 IU) IM | D6 |
|  |  | IT | D1 |
| HR | Block2 | Dexamethasone 20 mg/m^2^ PO/IV | D1-5 |
|  |  | Vindesine 3 mg/m^2^ IV | D1,6 |
|  |  | Methotrexate 5000 mg/m^2^ PI over 24 h | D1 |
|  |  | Ifosfamide 800 mg/m^2^ PI over 1 h | D2-4 (five doses, 12 h interval) |
|  |  | Daunorubicin 30 mg/m^2^ PI over 2 h | D5 |
|  |  | PEG-asparaginease 2500 mg/m^2^ (max. 3750 IU) IM | D6 |
|  |  | IT | D1 |
| HR | Block3 | Dexamethasone 10 mg/m^2^ PO/IV | D1-5 |
|  |  | Cytarabine 2000 mg/m^2^ IV PI over 3 h | D1-2 (four doses, 12 h interval) |
|  |  | Etoposide 100 mg/m^2^ PI over 24 h | D3-5 (five doses, 12 h interval) |
|  |  | PEG-asparaginease 2500 mg/m^2^ (max. 3750 IU) IM | D6 |
|  |  | IT | D1 |
| HR | Block1, Block2, Block3 | Repeat one cycle |  |
| IR/HR | Reinduction | Dexamethasone 10 mg/m^2^ PO | D1-7, D15-21 |
|  |  | Vincristine 1.5 mg/m^2^ (max. 2 mg) IV | D1,8,15,22 |
|  |  | Doxorubicin 30 mg/m^2^ PI over 1 h | D1,8,15,22 |
|  |  | PEG-asparaginase 2500 IU/m^2^ (max. 3750 IU) PI over 1 h | (IR only) D3,17,43 |
|  |  | Cyclophosphamide 1000 mg/m^2^ PI over 1 h | D29 |
|  |  | Cytarabine 75 mg/m^2^ IV | D31-34, D38-41 |
|  |  | 6-mercaptopurine 60 mg/m^2^ PO | D29-42 |
|  |  | IT | D1,15,31,38 |
| IR/HR | Maintenance | 6-mercaptopurine 50 mg/m^2^ PO daily |  |
|  |  | Methotrexate 20 mg/m^2^ PO weekly |  |
|  |  | Dexamethasone 10 mg/m^2^ PO for 5 days every 8 weeks |  |
|  |  | Vincristine 1.5 mg/m^2^ IV every 8 weeks |  |
|  |  | IT | Once every 4 weeks, 7 times |

Abbreviation: HR, high risk; IR, intermediate risk; IT, intrathecal; IV, intravenous push; max., maximum; PI, intravenous infusion; PO, by mouth; and SR, standard risk.
